# Pharmacokinetics and Pharmacodynamics of Colistin Combined With Isopropoxy Benzene Guanidine Against *mcr-1*-Positive *Salmonella* in an Intestinal Infection Model

**DOI:** 10.3389/fmicb.2022.907116

**Published:** 2022-05-20

**Authors:** Lingli Kong, Yixing Lu, Liuye Yang, Wanying Zhang, Beini Zuo, Xianfeng Peng, Zonghua Qin, Miao Li, Zhenling Zeng, Dongping Zeng

**Affiliations:** ^1^Guangdong Provincial Key Laboratory of Veterinary Pharmaceutics Development and Safety Evaluation, National Risk Assessment Laboratory for Antimicrobial Resistance of Animal Original Bacteria, College of Veterinary Medicine, South China Agricultural University, Guangzhou, China; ^2^Guangdong Laboratory for Lingnan Modern Agriculture, Guangzhou, China; ^3^Guangzhou Insighter Biotechnology Co., Ltd., Guangzhou, China; ^4^Division of Biochemical Toxicology, National Center for Toxicological Research, United States Food and Drug Administration, Jefferson, AR, United States

**Keywords:** colistin, isopropoxy benzene guanidine, pharmacokinetic/pharmacodynamic (PK/PD), intestinal infection model, *mcr-1*, *Salmonella*

## Abstract

Plasmid-borne colistin resistance mediated by *mcr-1* is a growing problem, which poses a serious challenge to the clinical application of colistin for Gram-negative bacterial infections. Drug combination is one of the effective strategies to treat colistin-resistant bacteria. Here, we found a guanidine compound, namely, isopropoxy benzene guanidine (IBG), which boosted the efficacy of colistin against *mcr-1*-positive *Salmonella*. This study aimed to develop a pharmacokinetics/pharmacodynamics (PK/PD) model by combining colistin with IBG against *mcr-1*-positive *Salmonella* in an intestinal infection model. Antibiotic susceptibility testing, checkerboard assays and time-kill curves were used to investigate the antibacterial activity of the synergistic activity of the combination. PK studies of colistin in the intestine were determined through oral gavage of single dose of 2, 4, 8, and 16 mg/kg of body weight in broilers with intestinal infection. On the contrary, PD studies were conducted over 24 h based on a single dose ranging from 2 to 16 mg/kg. The inhibitory effect *I*_*max*_ model was used for PK/PD modeling. The combination of colistin and IBG showed significant synergistic activity. The AUC_0−24*h*_/MIC index was used to evaluate the relationship between PK and PD, and the correlation was >0.9085. The AUC_0−24*h*_ /MIC targets in combination required to achieve the bacteriostatic action, 3-log_10_ kill, and 4-log_10_ kill of bacterial counts were 47.55, 865.87, and 1894.39, respectively. These results can facilitate the evaluation of the use of IBG as a potential colistin adjuvant in the treatment of intestinal diseases in broilers caused by colistin-resistant *Salmonella*.

## Introduction

Infections caused by *Salmonella* spp. are the commonly reported bacterial diseases in poultry, which may cause foodborne illnesses in humans, whereas children and young animals are more vulnerable to *Salmonella* infection (El-Sharkawy et al., 2017; Bailey et al., 2020). *Salmonella* enterica serovar Typhimurium (*S. typhimurium*) is one of the leading serovars responsible for global infectious diarrhea and foodborne disease outbreaks (Zhang et al., 2014). Colistin is prescribed for the treatment of enteric diseases, primarily in poultry and pigs submitted to intensive husbandry systems (Poirel et al., 2017). In recent years, the rapid transmission of colistin resistance mediated by plasmid-borne *mcr-1* gene has caused great concern (Liu et al., 2016; Wang et al., 2018; Elbediwi et al., 2019). The prevalence and spread of colistin resistant *S. typhimurium* in animals and food worldwide poses a challenge to the last line of defense, which could pose a serious threat to public health (Biswas et al., 2019; Elbediwi et al., 2020; Ramatla et al., 2022).

Given the importance of colistin in treating infections caused by multidrug-resistant Gram-negative bacteria, the development of resistance to this antibiotic has prompted new approaches to counter drug-resistant infections (Nang et al., 2021). Treatment with colistin in combination with other antibiotics or adjuvants is still effective against *mcr*-harboring bacterial infections (MacNair et al., 2018; Song et al., 2020). Substituted benzene guanidine compounds belonging to the amino-guanidine compound class have been widely used to treat a broad range of diseases, and they have become potential candidates for further structural modification of new drugs (Saczewski and Balewski, 2013; Rauf and Imtiaz-ud-Din, 2014; Liu et al., 2017). In previous study, we screened guanidine compound, that is, isopropoxy benzene guanidine (IBG) against drug-resistant *Enterococci* and *Staphylococcus aureus*, by disrupting the cell membranes (Zhang et al., 2019, 2021). Furthermore, we found that IBG can restore colistin susceptibility against *mcr-1*-positive *Salmonella*.

The purpose of this study was to determine the *in vitro* and *in vivo* activity of colistin combined with IBG, as well as determine the PK of colistin in an intestinal infection model infected with *mcr-1*-positive *Salmonella*. The surrogate PK/PD indexes were also analyzed to achieve various killing effects using the inhibitory effect *I*_*max*_ model. The discovery of IBG as a colistin adjuvant provides a potential therapeutic option against colistin-resistant *Salmonella* infections.

## Materials and Methods

### Antibiotic and Bacteria

Colistin sulfate (content 19,000 U/mg) powder was purchased from Meilun biotechnology Co., Ltd. (Dalian, China). Isopropoxy benzene guanidine (batch number20150506, content 99.9%) was synthesized by Guangzhou Insighter Biotechnology (< city>Guangzhou < /city>, China). Dimethyl sulfoxide (Dmreagent, Tianjing, China) was utilized as solvent to dissolve IBG. The organism was grown, sub-cultured and quantified in Mueller-Hinton Broth (Becton Dickinson, Sparks, MD, USA). Five isolates of *Salmonella*, including a standard strain (ATCC14028) obtained from the China Institute of Veterinary Drug Control and four *mcr-1*-positive clinical strains isolated from chickens, were used in this work. *E. coli* ATCC25922 strain was used as a reference strain for antibiotic susceptibility determination.

### Animals

Two weeks old healthy Sanhuang broilers (*n* = 240) weighting 100 ± 10 g were used in this study. Anal swabs were collected with sterile cotton swabs and cultured on chromogenic *Salmonella* agar to ensure that all broilers were found negative. Broilers were randomly selected to collect intestinal contents, then DNA was extracted using a fecal DNA extraction kit and PCR assay for the detection of *mcr-1*. This procedure was used to ensure that the broilers were not infected with *mcr-1*- positive *Salmonella*. All the broilers were not treated with an antibiotic before. They were housed in a climate-controlled environment under identical conditions with standard commercial diet and water supply ad libitum. All procedures were approved by the Institutional Animal Care and Use Committee of South China Agricultural University (approval number: 2021A019).

### Antimicrobial Combination Susceptibility Testing

The susceptibility of the selected *Salmonella* isolates and quality control isolate (ATCC 25922) to colistin and IBG in MH broth was investigated in accordance with the methods and quality control requirements recommended by the Clinical and Laboratory Standards Institute. Minimal inhibitory concentration (MIC) was the lowest concentration of colistin where visible bacterial growth was inhibited after 24 h of incubation. *E. coli* ATCC 25922 was used as the quality control strain to ensure the credibility of MICs tested. Susceptibility testing was performed in triplicate and germ-free controls (blank medium). The minimum bactericidal concentration (MBC) in MH broth was measured according to a previous report (Lei et al., 2017). The synergistic activity between colistin and IBG, and the fractional inhibitory concentration indices (FICI) were measured by checkerboard assays, based on the MIC of each drug (MacNair et al., 2018). In brief, 50 μL of broth was added to each well, and 50 μL of the indicated concentration of colistin was added into the first well of each line. This process was followed by multiple proportion dilution. Next, 50 μL of samples of different IBG concentrations was added to the dilution medium, and 100 μL of inoculum with a cell density of ~1 × 10^6^ colony-forming units (CFU)/mL was added to each well. The FICI was calculated follows:


(1) $$\begin{array}{r} FICI=MIC_{\left(\text{Colistinincombination}\right)}/MIC_{\left(\text{Colistin}\right)}\\ +MIC_{\left(\text{IBGincombination}\right)}/MIC_{\left(\text{IBG}\right)}. \end{array}$$

### *In vitro* Time-Killing Curves

After MIC determination, different concentrations of colistin were prepared in broth ranging from different doses before bacterial inoculation (10^6^ CFU/mL). Growth was checked with control. The tubes containing bacteria and different concentrations of colistin and IBG were incubated at 37°C. The bacterial count (CFU/mL) was determined for each tube after 0, 1, 2, 4, 6, 8, 12, and 24 h of incubation. In brief, 100 μL of culture was obtained for each time point, and serially diluted, and the colonies were counted the next morning. Each concentration test was performed in triplicate.

### Establishing *mcr-1*-Positive *Salmonella* Infection Model

Broilers were inoculated by oral gavage with 1 mL of *mcr-1*-positive Salmonella culture containing 109–1010 CFU/mL. Necropsies of broilers that had died from infection and of euthanized animals were investigated, and the ileum content samples of broilers were taken as previously described (Addwebi et al., 2014; Wang et al., 2020). DNA was extracted from ileum content samples using a fecal DNA extraction kit (G-Clone, Beijing, China) in accordance with the manufacturer's instructions.

The SYBR Green quantitative PCR (qPCR) assay for the detection of *mcr-1*-positive *Salmonella* was used in accordance with the manufacturer's instructions (Takara Biomedical Technology, Beijing). The cycling conditions were as follows: 95°C for 30 s and 40 cycles of 95°C for 5 s, 54°C for 30 s and 72°C for 1 min, followed by an increase from 65 to 97°C for melting analysis. The SYBR^®^ Green qPCR assay for *mcr-1* detection was performed using previously published primers *mcr-1*-F (5-AAAGACGCGGTACAAGCAAC-3) and *mcr-1*-R (5- GCTGAACATACACGGCACAG-3) (Li et al., 2017). The product size of the amplicon was 213 bp. Experiments were performed using Roche LightCycler 96 qPCR Real Time PCR system (Applied Roche), and data were analyzed using a Software LightCycler^®^ 96 SW 1.1 (Applied Roche).

### Pharmacokinetics of Colistin in a mcr-1-Positive *Salmonella* Infection Model

After 7-day acclimation, 200 infected broilers were randomly divided into five groups (*n* = 40/group). Pharmacokinetic analysis of colistin (2, 4, 8, and 16 mg/kg) alone and in combination (4 mg/kg colistin, 32 mg/kg IBG) was performed in *mcr-1*-positive *Salmonella* infection broilers following oral gavage. At 0.25, 0.5, 0.75, 1, 2, 4, 6, 8, 12, and 24 h after oral administration, four broilers per group were humanely killed and ileum contents (0.5 g) were sampled. The concentration of colistin in each ileum content sample was determined by validated liquid chromatography-tandem mass spectrometry (LC-MS/MS), with minor modifications. Briefly, intestinal contents (0.5 g) were extracted with 10 mL of 10% trichloroacetic acid and 10 mL of 4% lead acetate, homogenized for 1 min, ultrasonicated (30 min) and centrifuged (10,000 g, 10 min) to obtain supernatant. Thereafter, 2 mL of extraction solution was applied to HLB SPE cartridges, which were prewashed with 3 mL of methanol and 3 mL of water. The cartridges were then rinsed with 3 mL of 4% lead acetate. Afterward, the colistin was eluted with 6 mL of 20% methanol. The elute was evaporated to dryness under a gentle N_2_ stream at 40°C and the residue was dissolved in 0.5 mL of 0.1% formic acid for concentration analysis. The drug concentrations in intestinal contents were determined using HPLC-MS/MS. The calibration range was 0.25–5 μg/g. The intraday and interday precision levels varied from 2.1 to 9.8% and from 4.5 to 7.0%, respectively. The limit of detection (LOD) and limit of quantification (LOQ) were 0.10 and 0.25 μg/g, respectively.

The values of colistin intestinal content PK parameters in single-dose PK studies were obtained by non-compartmental analysis in Phoenix WinNonlin^®^ 8.2 (Certara, L.P., Princeton, NJ, USA).

### Pharmacodynamics of Colistin Combined With IBG in an Intestinal Infection Model

Pharmacodynamic analysis of combined colistin and IBG treatment in *mcr-1*-positive *Salmonella* infection broilers was performed at 3 h after administration of the inoculum. Afterward, 32 mg/kg of IBG was combined with 2, 4, 8, or 16 mg/kg of colistin, and colistin and IBG were also tested alone at 2, 4, 8, and 16 mg/kg (*n* = 4 for each group). The broilers were sacrificed after 24 h of therapy, and the intestinal content samples were aseptically removed and homogenized for CFU determination. Untreated control broilers were sacrificed before colistin treatment and at 24 h after treatment.

### Analysis of the PK/PD Relationship

PK/PD analysis was conducted using the inhibitory effect *I*_*max*_ model (Riviere and Toutain, 2011). This model is described by the following equation:


(2) $$\begin{array}{r} E=E_{0}-\frac{I_{\max}\cdot X}{IC_{50}+X} \end{array}$$

where *E* is the antibacterial effect, measured as change in log_10_CFU/g after 24 h of treatment, compared with the initial log_10_CFU/g in the untreated control broiler; *E*_0_ is the difference in the number of bacteria (log_10_CFU/g) in control samples between time 0 and 24 h. *I*_*max*_ is the maximum antimicrobial growth inhibition determined as the change in log_10_CFU/g after 24 h treatment with colistin combined with IBG. *X* is the predictive variable (expressed as AUC_0−24*h*_/MIC, C_max_/MIC, and %T>MIC), and *IC*_50_ is the *X* value producing 50% of the maximum antibacterial effect. The relationship between efficacy and the three PK/PD indices was determined by non-linear least-squares regression in a Phoenix WinNonlin^®^ PD model. The coefficient of determination (*R*^2^) was used to estimate the variance caused by regression for each PK/PD index.

The potential optimal dosage can be calculated using the following equation (Toutain et al., 2002, 2007):


(3) $$\begin{array}{r} Dose=\frac{\left(AUC/MIC\right)\cdot MIC\cdot CL}{fu\cdot F}, \end{array}$$

where dose (per day) is at steady state; CL is the clearance per day; AUC/MIC is the targeted endpoint for optimal efficacy in hours; MIC is the target pathogen; F is the bioavailability factor (from 0 to 1), and fu is the free fraction of the drug (from 0 to 1). In this study, fu = 1.

## Results

### *In vitro* Susceptibility Testing and Time-Kill Assays

MIC of colistin and IBG alone and in combination against the five *Salmonella* isolates in MH broth are listed in Table 1. We found that IBG could restore the efficiency of colistin against *mcr-1*-positive *Salmonella*. The MIC of colistin decreased 32-fold from 2 to 0.25 mg/L in the presence of 8 mg/L of IBG with an observed reduction in MIC below the Gram-negative clinical breakpoint.

**Table 1:** Antibacterial activity of colistin alone or in combination against *Salmonella* strains in MH broth.

**Strain**	**Characteristic**	**MIC (mg/L)**				**FICI**	**MBC (mg/L)**	
		**CST**	**IBG**	**CST (in combination)**	**IBG (in combination)**		**CST**	**IBG**
ATCC14028	*S. typhimurium*	0.5	>512	0.125	4	0.2578	1	>512
26FS14	*S. typhimurium*; *mcr-1*	2	>512	0.25	8	0.1563	4	>512
26FS26	*S. typhimurium*; *mcr-1*	2	>512	0.5	16	0.2812	4	>512
S235	*S. typhimurium*; *mcr-1*	2	>512	0.25	16	0.1875	4	>512
S226	*S. typhimurium*; *mcr-1*	2	>512	0.25	16	0.1875	4	>512

*CST, colistin; IBG, isopropoxy benzene guanidine*.

**Figure 1:**
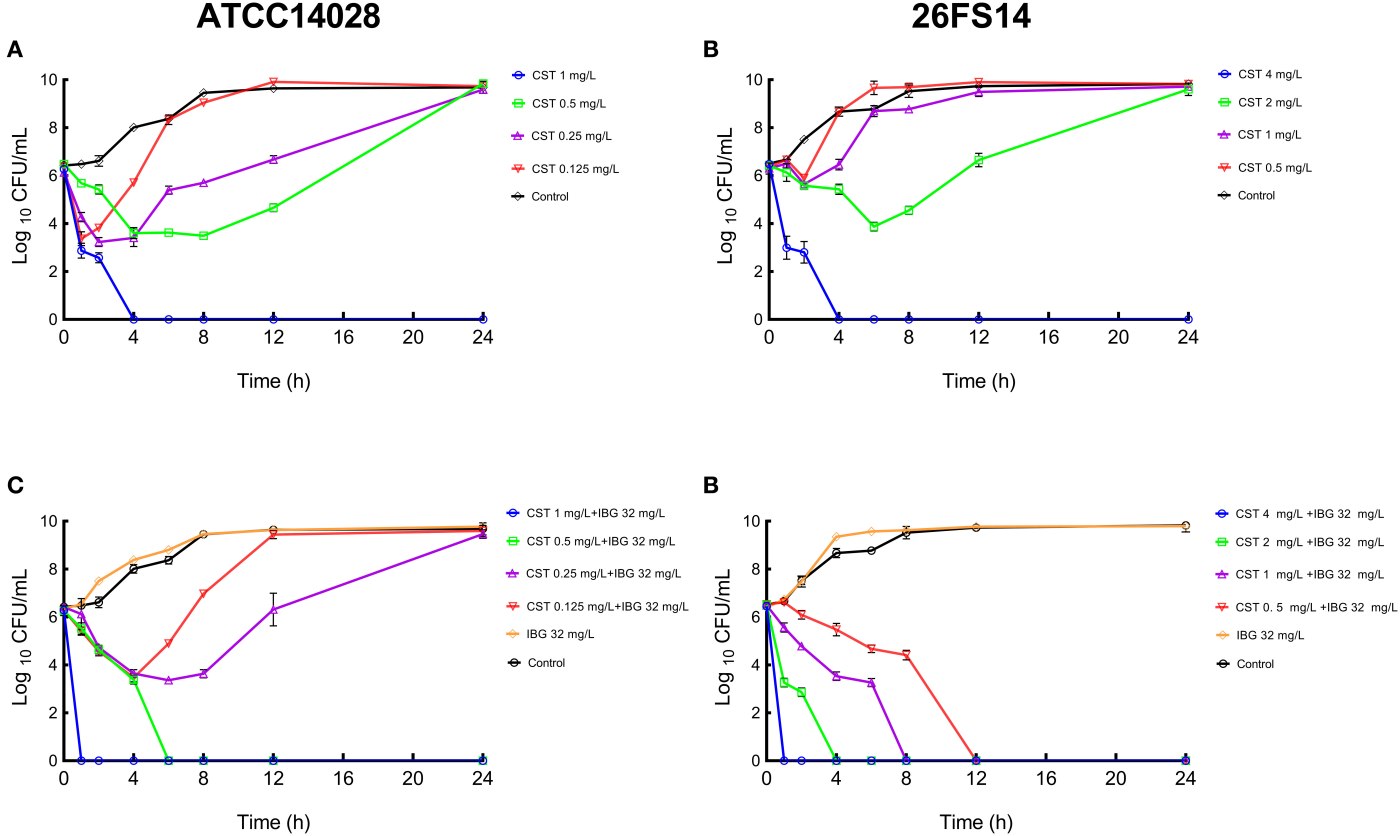
*In vitro* time-kill curve of colistin alone **(A,C)** and combined with IBG **(B,D)** against *Salmonella* ATCC14028 (left) and 26FS14 (right). A growth control without drug administration was included in all the tests. CST, colistin; IBG, isopropoxy benzene guanidine.

Base on the MIC values, a series of varying concentrations of colistin combined with IBG was prepared in broth to draw the *in vitro* time-killing curves against *Salmonella* (Figure 1). The profiles indicate a concentration-dependent killing characteristic of colistin, that is, increasing the drug concentration leads to rapid killing. When the drug concentration is lower than MIC, the bactericidal effect cannot be achieved within 24 h. Persisting inhibition of bacterial growth was observed when *mcr-1*-positive *Salmonella* was exposed to colistin at a concentration of 4 mg/L. However, the addition of 0.5 mg/L of colistin to 32 mg/L of IBG improved the antimicrobial activity of colistin. Either bactericidal effect or elimination of *mcr-1*-positive *Salmonella* could be obtained after 24 h of incubation. As observed in sensitivity tests, a synergistic antibacterial effect was observed in colistin-resistant and sensitive strains.

### Pharmacokinetics of Colistin in an Intestinal Infection Model

**Figure 2:**
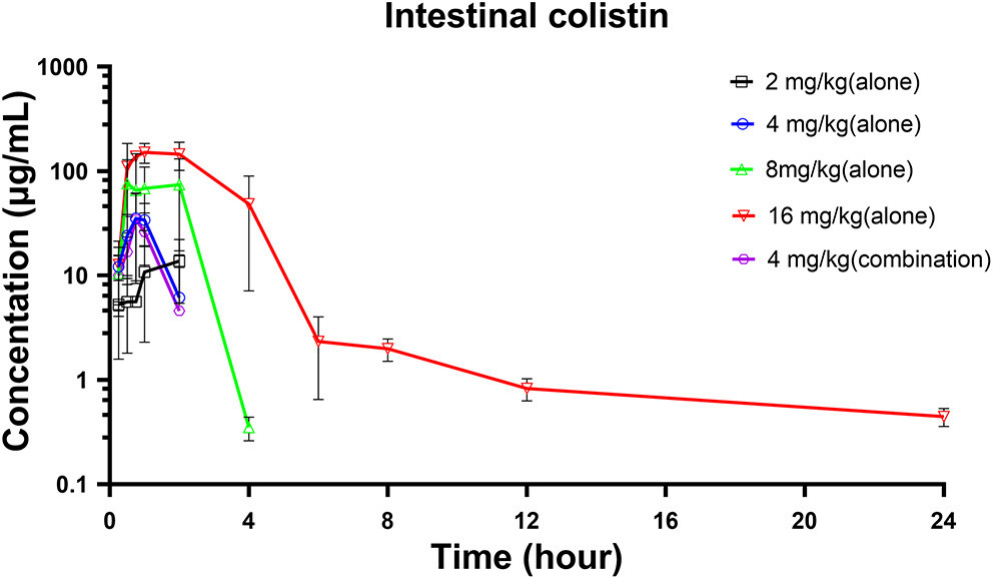
Intestinal colistin concentration-time courses in broilers infected with *mcr-1*-positive *Salmonella* following single gavage of 2, 4, 6, 8, and 16 mg/kg.

Colistin concentration-time courses in *Salmonella* infected broilers following single oral gavage at 2, 4, 8, and 16 mg/kg are shown in Figure 2. A good linearity of colistin was observed in the intestine (*R*^2^ ≥ 0.987 for C_max_ and AUC_0−24*h*_). The AUC_0−24*h*_ and C_max_ ranged from 31.58 to 494.40 mg·h/L and from 13.74 to 151.15 mg/L, respectively (Table 2). In addition, no significant difference in PK profiles was observed between colistin alone and in combination with IBG (*P* > 0.05).

**Table 2:** Pharmacokinetic parameters of intestinal colistin concentrations following single gavage (2–16 mg/kg) in *Salmonella* infected broiler.

**Parameter**	**Colistin**				**Colistin/IBG**
	**2**	**4**	**8**	**16**	**4**
C_max_ (mg/L)	13.74	34.37	75.92	151.15	35.29
AUC_0−24*h*_ (mg·h/L)	31.58	43.79	191.81	505.29	48.46
T>MIC (%)	15.11	16.48	16.62	99.98	13.87

*C_max_, the peak intestinal colistin concentration; AUC_0−24h_, the area under intestinal colistin concentration-time curve over 24 h*.

### Relationships Between PK/PD Indices and Antibacterial Activity

**Figure 3:**
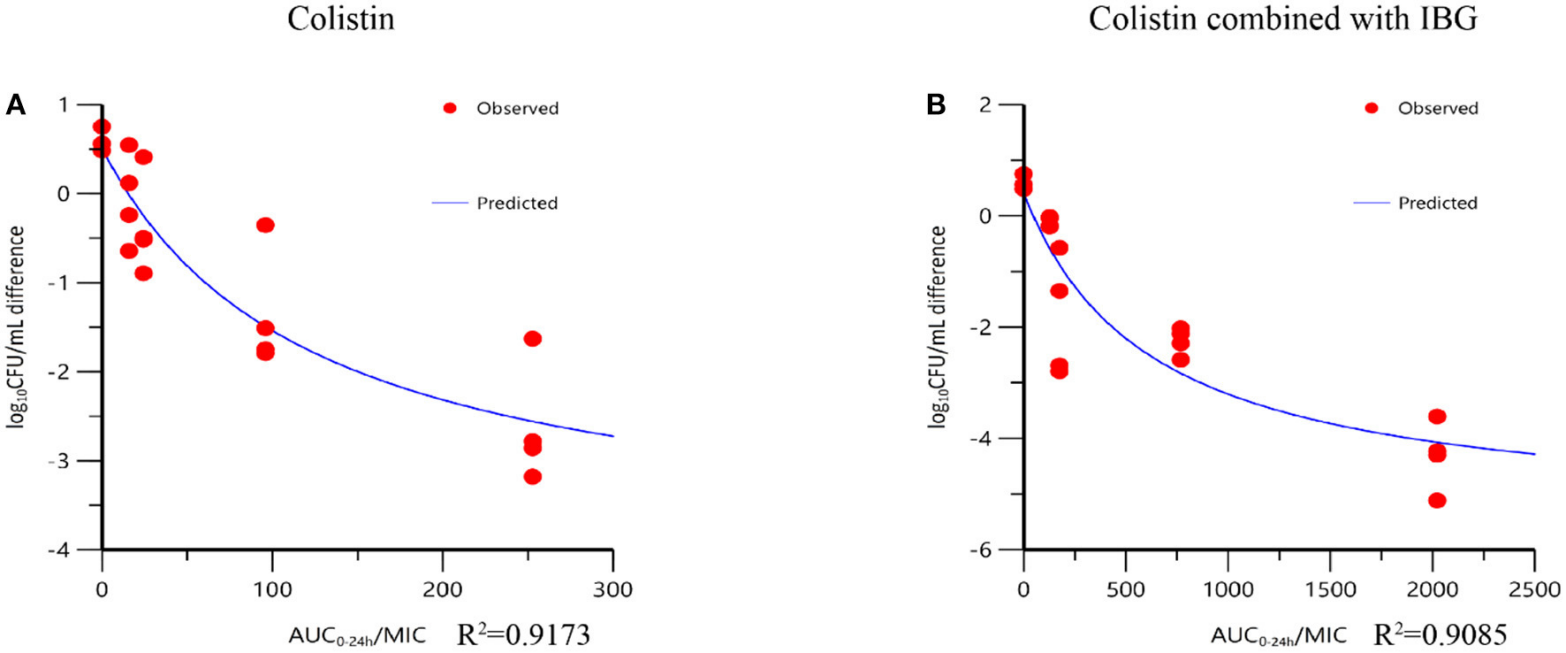
Relationships between the effect of colistin against *mcr-1*-positive *Salmonella* and the PK/PD indices AUC_0−24*h*_/MIC in the intestinal infection model. *R*^2^ is the coefficient of determination.

At the start of colistin combined with IBG therapy, bacterial burdens were 7.11 ± 0.23 log_10_CFU/g. In untreated animals, organisms grew at a rate of 0.59 ± 0.11 log_10_CFU/g over the next 24 h. The most effective colistin combined with IBG dosage regimens results in the reduction of bacterial number at the start of colistin combined with IBG treatment (4.31 ± 0.62 log_10_CFU/g). The inhibitory effect *I*_*max*_ model was utilized to simulate the relationships between AUC_0−24*h*_/MIC and *in vivo* antimicrobial efficacy. The relationships between the effect of colistin combined with IBG against *Salmonella* and each of the PK/PD indices in the intestinal infection model are shown in Figure 3. The AUC_0−24*h*_/MIC ratios required for various efficacy targets in the intestinal infection model are shown in Table 3.

**Table 3:** PK/PD parameter estimates for the AUC_0−24*h*_/MIC index and AUC_0−24*h*_/MIC values required for various antibacterial effects.

**Parameter**	**Colistin**	**Colistin/IBG**
*E_0_* (log_10_CFU/lung)	0.51	0.41
*I_*max*_* (log_10_CFU/lung)	4.55	5.85
*IC_50_*	122.19	619.45
AUC_0−24*h*_/MIC for bacteriostatic action	15.62	47.55
AUC_0−24*h*_/MIC for 1 log_10_ kill	60.66	197.70
AUC_0−24*h*_/MIC for 2 log_10_ kill	150.15	435.44
AUC_0−24*h*_/MIC for 3 log_10_ kill	–	865.87
AUC_0−24*h*_/MIC for 4 log_10_ kill	–	1894.39

### Dose Determination

The doses required to reach stasis, a 1 × l og_10_ kill, and 2 × log_10_ kill effect for colistin monotherapy were 1.94, 7.54, and 18.68 mg/kg, respectively. For 32 mg/kg of IBG-based combinations, the required dose was reduced by 2-fold, and 0.74, 3.07, and 6.77 mg/kg of colistin achieved the same effect. For colistin resistant strains, the doses in this experiment could not achieve 3 × log_10_ kill and 4 × log_10_ kill effect for colistin monotherapy. For 32 mg/kg IBG-based combinations, the doses required to reach 3 × and 4 × log10 kill effect were 13.46, and 29.45 mg/kg, respectively.

## Discussion

Colistin is considered as the last-line treatment regimens against carbapenem-resistant *Enterobacteriaceae* (Nang et al., 2021). The emergence and spread of plasmid-mediated resistance gene *mcr-1* seriously threatens the effectiveness of colistin, which pose a severe threat to public health (Shi et al., 2020). Colistin may accelerate the transmission when *mcr-1*-carrying bacteria reach the gut in broilers (Miguela-Villoldo et al., 2021). Treatment failures with colistin monotherapy have promoted the search for combination therapy. New adjuvants can restore the effectiveness of existing antibiotics, and they have been considered as a cost-effective strategy for fighting resistant bacteria (Liu et al., 2019). As guanidine had strong organic bases and presented hydrophilic, guanidine compounds have been used to treat a variety of diseases and discovered as new promising drugs (Saczewski and Balewski, 2013; Massimba-Dibama et al., 2015). Liu revealed the potential of metformin as a novel tetracyclines adjuvant restored against multidrug resistant bacteria (Liu et al., 2020). Robenidine combined with polymyxin B non-apeptide had synergistic antibacterial activities against *Pseudomonas aeruginosa, Escherichia coli* and *Klebsiella pneumoniae* (Khazandi et al., 2019). IBG is a substituted benzene guanidine derivative, which showed a promising safety profile with human lung epithelial cells (A549) had cytotoxicity of IC_50_ 28 μg/mL, and it was well-tolerated by erythrocytes in mice, with HC_50_ of >400 μg/mL (Zhang et al., 2019). In addition, IBG supplementation effectively improved the average daily gain and reduced diarrhea rate of broilers without adverse reactions (Xiao et al., 2018). In the present study, we found that IBG restored the antibacterial activity of colistin against *mcr-1*-positive *Salmonella*. Interestingly, synergistic antimicrobial effects were observed against colistin-resistance and sensitive isolates, when colistin was combined with IBG. The MIC of colistin decreased 32-fold from 2 to 0.25 mg/L in the presence of 8 mg/L of IBG with an observed reduction in MIC below the Gram-negative clinical breakpoint. Re-growth of *mcr-1*-positive *Salmonella* was not observed within 24 h after exposure to 0.5 or 1 mg/L of colistin combined with 32 mg/L of IBG. These results indicated that IBG could reduce the dose of colistin and reduce the occurrence of adverse reactions.

Although the PK of colistin in the plasma of mice, rat, and sheep after administration has already been investigated (Landersdorfer et al., 2017; Sivanesan et al., 2017; Bouchene et al., 2018), reports concerning the PK in the intestinal contents are few. Colistin is hardly absorbed after oral administration and is excreted through the gastrointestinal tract in animals (Rhouma et al., 2016). Oral administration and drinking water are the main drug delivery routes in livestock and poultry breeding. After oral administration, the plasma concentration of colistin is negligible, and it does not provide a valid quantification of the gastrointestinal antibacterial effect (Guyonnet et al., 2010). Determining colistin PK characteristics in the digestive tract of livestock and poultry is necessary to defend colistin as the last-line antibiotic. In this investigation, we described the extraction of colistin from broiler intestinal ileum content using SPE purification and LC-MS/MS to quantify colistin for PK/PD investigations. The concentration of colistin in ileum content increased gradually after gavage, followed by a rapid decline as poultry chyme was transported. At doses <16 mg/kg, no colistin was detectable in ileum samples after 4 h. In agreement with our study, Mead et al. (2021) demonstrated a rapid elimination, with colistin levels below the LOQ within 4 h of dosing cessation after oral administration of colistin at dose rates of 75,000 I.U./kg in chicken.

In this study, the inhibitory effect *I*_*max*_ model was used for PK/PD modeling. The result showed that the PK/PD index of AUC_0−24*h*_/MIC(*R*^2^ > 0.9085) had a strong correlation with antibacterial activity in the intestinal infection model. In the present study, a single dose of 2–16 mg/kg of colistin every 24 h has not significant effect on broilers infected with *mcr-1* positive *Salmonella*. Increased antimicrobial activity was observed by supplementing colistin with IBG in intestinal model broiler. Based on the *in vivo* antimicrobial effects, treatment with ≤ 16 mg/kg of colistin could not achieve 3 × log_10_ kill effect for colistin monotherapy against *mcr-1*-positive *Salmonella*. An evident bactericidal effect (>4-log_10_ decrease in bacterial burden) was achieved by 32 mg/kg of IBG plus 16 mg/kg of colistin. The AUC_0−24*h*_/MIC targets required to achieve bacteriostatic action, a 3-log_10_ kill and 4-log_10_ kill of bacterial counts were 34.33,348.95, and 559.86, respectively.

Colistin combined with other antibiotics or adjuvants can effectively treat colistin-resistant bacteria infection, and also reduce the dose of colistin to reduce toxic and side effects. Yi et al. (2022) report the synergistic activity of tetrandrine combined with colistin against the *mcr-1*-positive *Salmonella* both *in vitro* and *in vivo*. The tetrandrine dramatically undermines the efflux pump and PMF function of colistin-resistant bacteria, and enhanced the membrane damage ability of colistin. confirm the synergistic effects of osthole (OST) as a potent MCR-1 inhibitor against *mcr-1*-positive colistin-resistant *Enterobacteria* both *in vitro* and *in vivo* (Nang et al., 2021). With regard to the mechanism of action, IBG was used to fight drug-resistant *Enterococci* and *Staphylococcus aureus* by disrupting the cell membranes (Zhang et al., 2019, 2021). Colistin may have damaged the outer membrane of *Salmonella*, and IBG further plays a synergistic role by destroying cell membrane potential and cytoplasmic membrane integrity. Changes in the transcriptome and protein content of *Salmonella* following treatment with drugs should be examined in the future to investigate the potential mechanism of the combined activity of colistin and IBG against *Salmonella* strains.

This study is the first to demonstrate the use of IBG as a colistin adjuvant against *mcr-1*-positive *Salmonella* in broilers. We determined the AUC_0−24*h*_/MIC targets in the intestine to achieve various magnitudes of bacterial kill. Our study provides a new direction for the optimized clinical use of colistin against infections caused by *mcr-1*-positive *Salmonella*.

## Data Availability Statement

The original contributions presented in the study are included in the article/supplementary material, further inquiries can be directed to the corresponding author/s.

## Ethics Statement

The animal study was reviewed and approved by all procedures were approved by the Institutional Animal Care and Use Committee of South China Agricultural University (approval number: 2021A019).

## Author Contributions

DZ and ZZ conceived this study and participated in its design and coordination. LK and YL designed the experiments and drafted the manuscript. YL, WZ, and BZ carried out the *in vivo* animal experiments. XP and ZQ worked on the synthesis of compound IBG. DZ, ZZ, LK, ML, and YL conducted the PK/PD analysis. All authors read and approved the final manuscript.

## Funding

This work was supported by Local Innovative and Research Teams Project of Guangdong Pearl River Talents Program (grant number 2019BT02N054).

## Author Disclaimer

The opinions expressed in this manuscript do not necessarily represent those of the U.S. Food and Drug Administration.

## Conflict of Interest

XP and ZQ were employed by Guangzhou Insighter Biotechnology Co., Ltd. The remaining authors declare that the research was conducted in the absence of any commercial or financial relationships that could be construed as a potential conflict of interest.

## Publisher's Note

All claims expressed in this article are solely those of the authors and do not necessarily represent those of their affiliated organizations, or those of the publisher, the editors and the reviewers. Any product that may be evaluated in this article, or claim that may be made by its manufacturer, is not guaranteed or endorsed by the publisher.
